# How mountains and elevations shape the spatial distribution of beetles in Peninsular Malaysia

**DOI:** 10.1038/s41598-021-84965-5

**Published:** 2021-03-11

**Authors:** Muneeb M. Musthafa, Fauziah Abdullah, Ana Paola Martínez-Falcón, Mark de Bruyn

**Affiliations:** 1 Department of Biosystems Technology, Faculty of Technology, South Eastern University of Sri Lanka, University Park, Oluvil, 32360 Sri Lanka; 2 Institute of Biological Science, Faculty of Science, University of Malaya, 50603 Kuala Lumpur, Malaysia; 3 Centro de Investigaciones Biológicas, Instituto de Ciencias Básicas e Ingeniería, Universidad Autónoma del Estado de Hidalgo, Carretera Pachuca-Tulancingo km 4.5, C.P. 42184 Mineral de La Reforma, Hidalgo Mexico; 4 School of Life and Environmental Sciences, The University of Sydney, Camperdown, NSW 2006 Australia

**Keywords:** Ecology, Ecology, Environmental sciences

## Abstract

This study was conducted to assess the spatial distribution of beetles in mountain ecosystems and their elevational diversity. Malaise, pitfall and light traps were used to collect beetles from nine different mountains in Malaysia from September 2014 to September 2016, where from Gunung Angsi, Gunung Belumut, Gunung Basor and Gunung Tebu samples were collected at 500 m and 1000 m (above sea level) elevations, while beetles were sampled at 500 m, 1000 m and 1500 masl from Gunung Benom, Gunung Inas, Cameron Highland, Gunung Besar Hantu and Gunung Basor. In this study, 9628 beetles belonging to 879 different species were collected with highest representation from family Staphylinidae and Carabidae. Chamah Highland had the highest beetle diversity followed by Gunung Benom, Gunung Inas, Cameron Highland, Gunung Belumut, and Gunung Basor. Chamah Highland was different to all mountains on abundance and species richness. The highest species richness was observed at 1000 m, followed by 500 m and 1500 m. We identified characteristic species associated with habitat conditions at Gunung Benoum and Gunung Inas mountains, according to INDVAL values. The beetle diversity of the sampled mountains showed multiple alpha and beta patterns according to type of mountain ecosystem and elevation, providing guidelines for the scientific community to underpin conservation efforts in Malaysia.

## Introduction

Major tropical mountain ecosystems are exposed to greater vulnerability than ever before, with elevational responses of species providing opportunities to forecast the ecological consequences of global change on montane ecosystems^1,2^. Mountain regions/ranges harbor high levels of endemic habitat specialist taxa, shaped by unique environmental factors and relatively limited range shifts^2,3^. Among the environmental factors shaping these communities, elevation exerts a dominant influence on species diversity, driven primarily by changes in temperature and precipitation^4^. Moreover, every mountain has its own history, with specific geological and geographical drivers, combined with anthropogenic influences, and biotic and abiotic interactions^5^, producing complex diversity distribution patterns^6,7^. The patterns and distribution of different species and species groups along elevations in montane ecosystems differ widely according to past and present environmental changes^8–10^.

Tropical mountains provide a good platform to study species responses to ecological changes across elevational gradients^11–14^. Montane ecosystems are widespread in Malaysia covering around 7% of the total land area according to the Economic Planning Unit (2016)^15^. The major mountains in Malaysia are located at the middle of a ridge running from Pahang to Kelantan States. According to Sodhi and Brook^16^, 23% of the original tropical montane forests have been lost or degraded in Malaysia, while just 9% (216,300 ha) of the remaining are listed as protected. When considering the roles of Malaysian cloud forests and looming threats to them, it is vital to improve current cloud forest protection in Malaysia^17,18^. Malaysian cloud forests are fragile forest ecosystem facing increasing threats in the form of anthropogenic disturbances and global warming^19–21^, which can be the driving force for the loss of these pristine habitats and their endemic fauna and flora^21^.

Montane forest ecosystems provide a good foundation to study biogeographic variation in the determinants of community structure, as their abiotic environment often varies dramatically in predictable ways along elevational gradients^22^. Montane forests are also often confined to small geographic areas, which are prone to topographical fragmentation, exposing these ecosystems to further vulnerability^21,23^. Finally, studies on tropical montane cloud forest ecosystems have contributed just 5% of biodiversity research in Southeast Asia, in comparison to 74% for lowland forests over the last two decades^2^.

Here, we hypothesize that the spatial distribution of beetles is shaped by mountain ecosystems and their elevational gradients, due to the fact that beetles play variety of roles in any ecosystem such as, pollinators, mediating in nutrient recycling, decaying plant and animal materials, parasites, seed dispersal and ecological maintenance in an ecosystem. Moreover, some beetles are considered as very good bio-indicators around the world since they are very sensitive to environmental changes. Thus, the specific aims of this study were: (1) to compare beetle diversity between the studied mountains, elevations and different sampling strategies. We predict that mid-elevation sites will comprise greater diversity, based on a number of studies^24–26^. Moreover, each mountain’s biodiversity has been shaped by a number of mechanisms, including higher speciation rates combined with greater coexistence and persistence of lineages influenced by long-term climatic changes interacting with topographically dynamic landscapes^27–30^ (2) to explore if any beetle species can be considered indicator species of each of the mountains and elevations. We hypothesize that indicator species can be a useful tool to assess long-term biodiversity changes across space; (3) to compare compositional similarity and beta diversity among mountains and elevations: given the unique climatic and topographic conditions on each mountain, we expect high species replacement among mountains.

## Results

This study collected 9628 beetles representing 879 different species with highest representation from family Staphylinidae and Carabidae, with *Paederus* sp1 being the most abundant beetle species collected (n = 493), followed by *Eleusis kraatzi* (n = 324). The beetle family-wise results showed that, there are ten families collected with more 200 individuals (Table 1). Only seven species was collected from all nine sampled mountains (*Anomala grandis*, *Hoplocerambyx spinicornis*, *Aulacophora intermedia*, *Nodostoma brevicolle*, *Metialma* sp1 and *Pachyderes macrothorax*). The greatest number of beetles were collected from Gunung Inas (n = 1727), closely followed by Chamah Highland (n = 1695), while the lowest number of individuals was collected from Gunung Tebu (n = 552).

**Table 1:** Top ten beetle families collected from the sampled mountains.

Beetle family	Number of individuals	Percentage
Staphylinidae	2545	26.43
Chrysomelidae	1424	14.79
Carabidae	883	9.17
Curculionoidea	875	9.09
Scarabaeidae	636	6.61
Bostrichidae	275	2.86
Nitidulidae	271	2.81
Coccinellidae	241	2.50
Cerambycidae	201	2.09
Elateridae	209	2.17

### Biodiversity patterns

**Figure 1:**
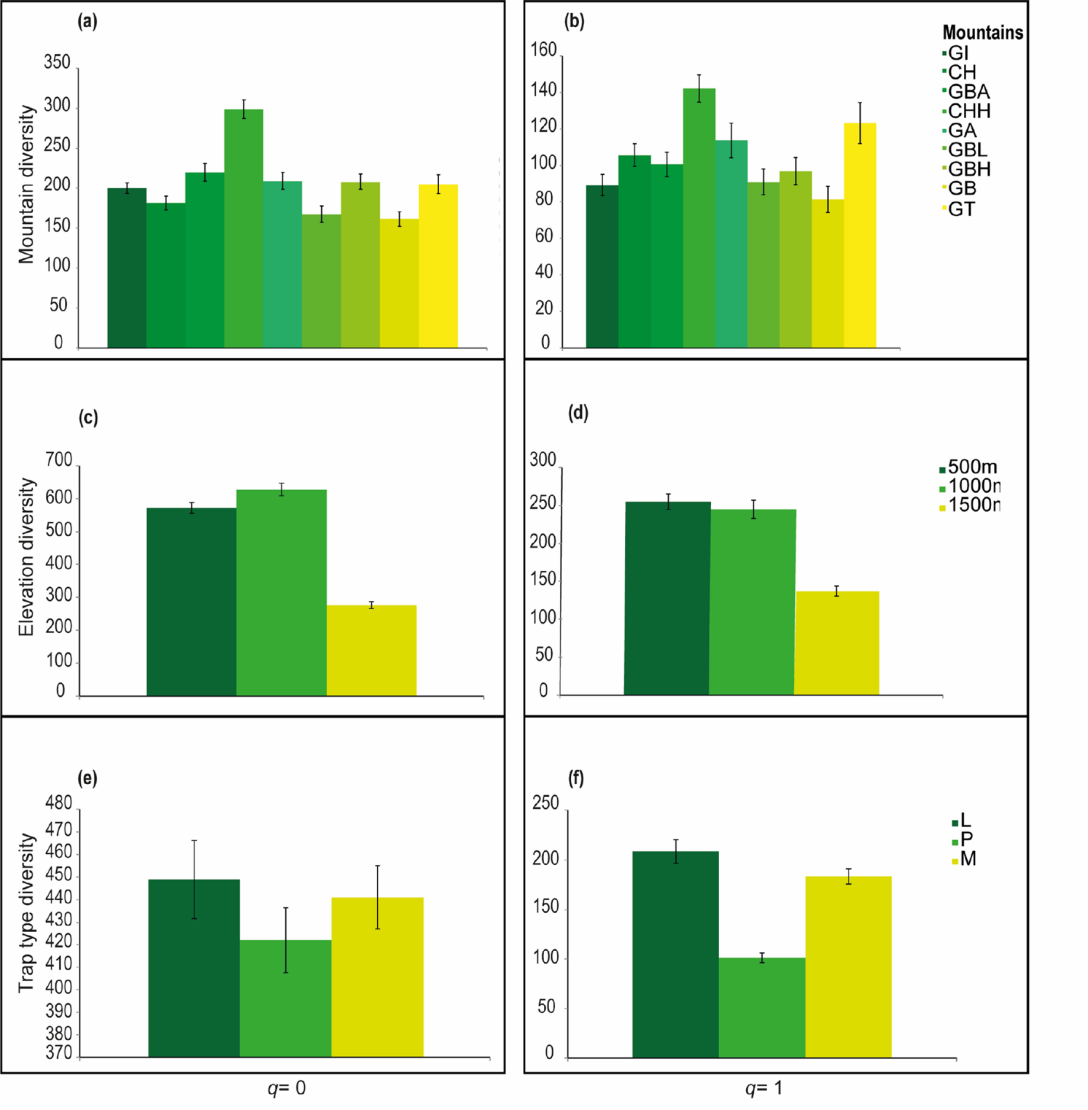
Hill number for the sampled mountains with 95% confidence intervals. Species richness (q = 0) and species diversity (q = 1) were calculated accounting for mountains, (*GI *Gunung Inas, *CH* Cameron Highlands, *GBA* Gunung Basor, *CHH* Chamah Highlands, *GA* Gunung Angsi, *GBL* Gunung Belumut, *GBH* Gunung Besar Hantu, *GB* Gunung Benom, *GT* Gunung Tebu), elevations, and trap typology (*L* light trap, *P* pitfall trap, *M* Malaise trap).

Comparing values for *q* = 0, Chamah Highland was significantly different from the other mountains. The second highest species richness was from Gunung Benom, which was higher than Gunung Inas, Cameron Highland, Gunung Belumut, and Gunung Basor, but not significantly different to Gunung Ansi, Gunung Besar Hantu, and Gunung Tebu (Fig. 1a,b). Taking into account *q* = 0 and *q* = 1 Chamah Highland’s beetle fauna was significantly different compared with all mountains except Gunung Tebu, which was also placed second in terms of *q* = 1 value (^*1*^*D* = 123), and also differed from the other mountains, except for Gunung Ansi (^*1*^*D* = 114) (according to confidence intervals). For *q* = 0 and *q* = 1, Gunung Basor had the lowest values (^*0*^*D* = 161, ^*1*^*D* = 81), but for *q* = 0, was different from all mountains, except Gunung Belumut (^*0*^*D* = 167), and for *q* = 1, was different from all mountains, except for Gunung Inas (^*1*^*D* = 89) (Fig. 1a,b).

For elevational differences according to *q* = 0, the highest species richness was at 1000 m (^*0*^*D* = 628), secondly for 500 m (^*0*^*D* = 572), with significant differences, while 1500 m had the lowest value (^*0*^*D* = 276) (Fig. 1c). For *q* = 1, the species diversity patterns changed, 500 m and 1000 m not significantly different (^*1*^*D* = 255, ^*1*^*D* = 245) (Fig. 1d), but 1500 m was, and still had the lowest species diversity value (^*1*^*D* = 137) (Fig. 1d).

Sample coverage for all the trapping methods were displayed a value above 0.9 (light trap 0.91; pitfall trap 0.97; Malaise trap 0.96). For trap diversity patterns, we did not detect differences for species richness (*q* = 0) ^*0*^*D* = 449, ^*0*^*D* = 422, ^*0*^*D* = 441) (Fig. 1e), but we found significant differences using *q* = 1, with light traps showing the highest diversity (^*1*^*D* = 209) and pitfall traps the lowest (^*1*^*D* = 101) (Fig. 1f).

Only the Gunung Benom and Gunung Inas Mountains had characteristic beetle species associated with significant indicator values, where *Coccotrypes variabilis* comprised 65% of the IndVal value (*p* = 0.001) at Gunung Benom. Both *Canthydrus haagi* with 60% (*p* = 0.001) and *Dischisus notulatus* with 54% (*p* = 0.001) showed considerable indicator values at Gunung Inas and considered as indicator species for these mountains. For elevational gradients, none of the beetle species had a significant IndVal value.

### Compositional similarity and beta diversity

**Figure 2:**
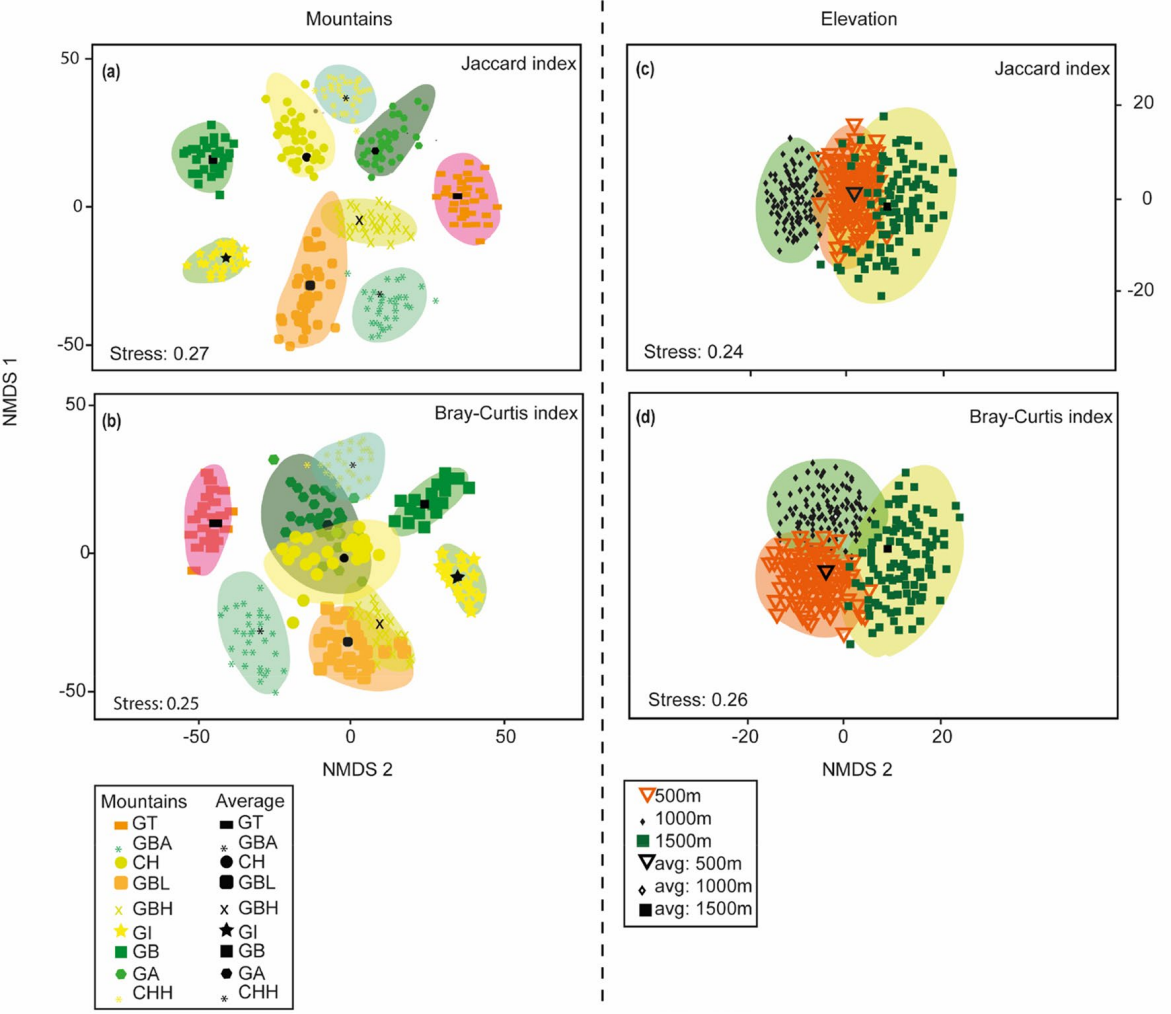
NMDS ordination for mountains and elevations as defined by using Jaccard index (**a**,**c**) and Bray–Curtis index (**b**,**d**) for beetles’ species abundance in Malaysia.

Differences in species composition were detected for mountains according to the Jaccard index (*F*_pseudo_ = 3.30, *df* = 8, *p* = 0.001) (Fig. 2a) and the Bray–Curtis index (*F*_pseudo_ = 3.07, *df* = 8, *p* = 0.001). All the pairwise comparisons with Jaccard values had significant differences correspondingly to NMDS (*p* < 0.001) (Table 2). Even though the MDS (Fig. 2b) suggests that Gunung Belumut (GBL) and Gunung Besar Hantu (GBH) overlap, the *p* value of the Bray–Curtis index showed significant differences between them. For elevation, significant differences were evident using the Jaccard index for the overall model using the Jaccard index (*F*_pseudo_ = 1.43, *df* = 2, *p* = 0.001), but pairwise comparisons did not detect differences among any elevation (Fig. 2c). For Bray–Curtis values we did not find significant differences (*F*_pseudo_ = 1.30, *df* = 2, *p* = 0.05) (Fig. 2d). The discrepancy between NMDS and PERMANOVA may be associated to the relatively high value of NMDS stress that is considered good below 0.2.

**Table 2:** Beta diversity of all mountain pairs and the values of its two components (turnover and nestedness).

Pairs	Total dissimilarity	Turnover	Nestedness
Gung Inas—Cameron Highland	0.67	0.6062718	0.06620209
Gung Inas—Gunung Benom	0.73	0.6706949	0.06042296
Gung Inas—Chamah Highland	0.71	0.4547804	0.25581395
Gung Inas—Gunung Angsi	0.82	0.7988506	0.02586207
Gung Inas—Gunung Belumut	0.81	0.7138264	0.10610933
Gung Inas—Gunung Besar Hantu	0.8	0.7764706	0.02352941
Gung Inas—Gunung Basor	0.81	0.6842105	0.12828947
Gung Inas—Gunung Tebu	0.8	0.7869822	0.0147929
Cameron Highland—Gunung Benom	0.68	0.557377	0.12786885
Cameron Highland—Chamah Highland	0.72	0.4053333	0.31466667
Cameron Highland—Gunung Angsi	0.75	0.6687898	0.08917198
Cameron Highland—Gunung Belumut	0.79	0.7430556	0.04861111
Cameron Highland—Gunung Besar Hantu	0.77	0.6918239	0.08490566
Cameron Highland—Gunung Basor	0.79	0.7208481	0.07067138
Cameron Highland—Gunung Tebu	0.85	0.7797619	0.07142857
Gunung Benom—Chamah Highland	0.74	0.5485437	0.19174757
Gunung Benom—Gunung Angsi	0.8	0.7709497	0.03072626
Gunung Benom—Gunung Belumut	0.76	0.5987261	0.16878981
Gunung Benom—Gunung Besar Hantu	0.77	0.7356322	0.03448276
Gunung Benom—Gunung Basor	0.79	0.6119874	0.18611987
Gunung Benom—Gunung Tebu	0.89	0.8571429	0.03896104
Chamah Highland—Gunung Angsi	0.71	0.489899	0.22727273
Chamah Highland—Gunung Belumut	0.82	0.4974874	0.33165829
Chamah Highland—Gunung Besar Hantu	0.82	0.612529	0.21113689
Chamah Highland—Gunung Basor	0.83	0.4822335	0.35025381
Chamah Highland—Gunung Tebu	0.9	0.70282	0.20390456
Gunung Angsi—Gunung Belumut	0.76	0.625	0.1381579
Gunung Angsi—Gunung Besar Hantu	0.8	0.8022923	0.00286533
Gunung Angsi—Gunung Basor	0.78	0.625	0.15789474
Gunung Angsi—Gunung Tebu	0.89	0.8882979	0.0106383
Gunung Belumut—Gunung Besar Hantu	0.71	0.5704467	0.14089347
Gunung Belumut—Gunung Basor	0.8	0.7854545	0.02181818
Gunung Belumut—Gunung Tebu	0.87	0.7613293	0.11480363
Gunung Besar Hantu—Gunung Basor	0.79	0.6405229	0.15359477
Gunung Besar Hantu—Gunung Tebu	0.88	0.8726287	0.00813008
Gunung Basor—Gunung Tebu	0.87	0.7423313	0.13496933

**Figure 3:**
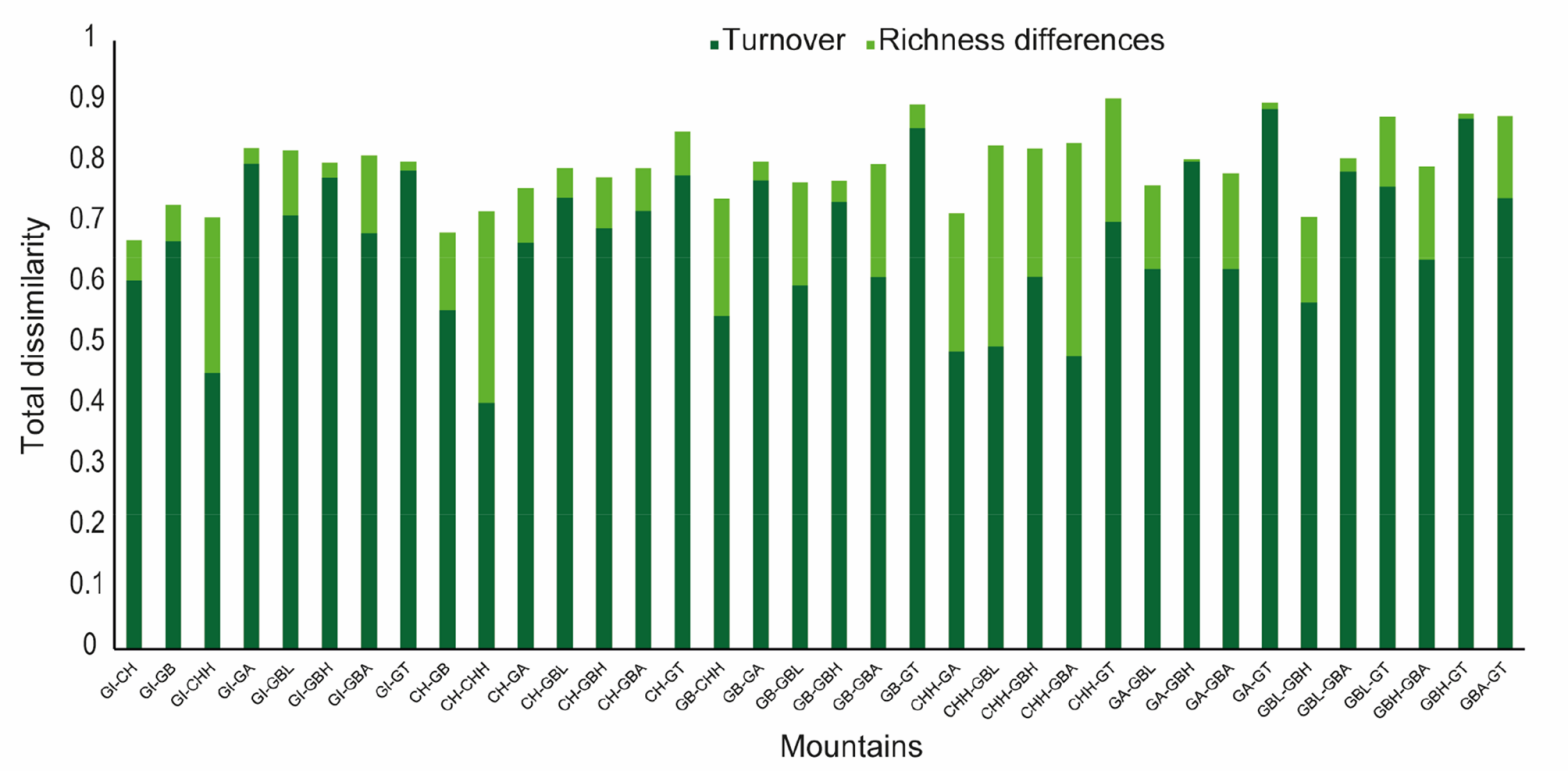
Total dissimilarity in species composition (beta diversity) and its composition (turnover and nestedness) between pairs of nine mountains in Malaysia.

Beta diversity was high (80% on average) among all the mountains, while species replacement was more important than turnover and nestedness for the two components of beta diversity calculated with incidence data (Fig. 3). However, there are pairs of communities including CHH Mountains in which nestedness was almost equal to turnover (Fig. 3). These mountains present unique change in species composition in the CHH Mountain is not the result of nestedness; they present unique species that are not shared with the other mountains (53.4% of the total species were only collected from a single mountain) such as *Erystus villicus, Melanotus hapalesus, Exomala orientalis, Apolecta asperiscollis*, etc.

## Discussion

Beetle species richness, diversity, indicator species and trapping methods showed different patterns at the nine sampled Peninsular Malaysian Mountains in this study^28–30^. Of the sampled mountains, Chamah Highland showed the highest beetle diversity and richness, likely a result of the remote and secluded nature of this mountain, presence of dense forest, and lack of anthropogenic disturbances^31,32^. Moreover, the Chamah Highland area is predominantly comprised of granitic rocks with biologically rich mountainous terrain, rivers, waterfalls, and rock outcrops^31^. Chamah Highland is one of the largest montane forests in Peninsular Malaysia, thus it is especially important for flora and fauna conservation. In addition to biotic elements, abiotic factors should be further investigated. While this mountain is relatively undisturbed, the study by Aweng et al.^33^ on benthic macroinvertebrates of rivers in the Chamah Highland reports some pollution. Therefore, this mountain is important from a biogeographical perspective, and should be studied in detail by the scientific community. Gunung Benom showed the second highest species richness (*q*0) among the sampled mountains, which is also reported to be pristine in nature and similarly undisturbed by human influences, further supporting the hypothesis that lack of anthropogenic impact coincides with greater biodiversity.

Gunung Tebu showed high species richness, where this forest reserve contains valuable tropical flora with timber species belonging to families Euphorbiaceae and Dipterocarpaceae contributing towards a large diversity of trees species^34^. These perennial plants likely accommodate an increased number of beetle species. Further, the presence of a number of interesting insect species^35^ and the relatively healthy conditions of Gunung Tebu Forest Reserve make it vital to direct conservation efforts to maintaining this ecosystem.

Beetle species richness showed a mid-elevational (1000 m) peak, whereas diversity peaked at lower elevations (500 m), closely followed by mid-elevation. Our data thus support our central hypothesis that mid-elevations will accommodate greater species richness^36^. A mid-elevational peak in diversity, and decreasing trends in richness, with increasing altitude are widely reported patterns across diverse plant and animal taxa^37–40^.

For ecological diversity (*q* = 1), the middle and low elevations had almost the same values, this means that taking into account abundance, both elevations have the same evenness, while the structure of beetle communities at 1500 m elevations had an assemblage of species dominated by *Paederus* sp1. Even though *Paederus* sp1. was also the most important species at the other elevations, at 1500 m, abundance of this species was great in contrast to the rest of the species found at this elevation. For the other elevations, less dramatic differences in species abundance were observed, for example, at 500 m, the second most abundant species was *Eleusis kraatzi*, with 152 individuals, while 157 *Paederus* sp1. individuals were sampled at this elevation. The three different types of traps also impacted on differences in beetles captured in relation to species diversity, richness, and trap attractiveness to different beetles. These traps are widely used for passive sampling of beetles; however, all these trapping methods are complementary to each other^41^. In this study, the type of trap did not affect species richness capture (*q* = 0), however for abundance data, we detected increased capture rates with light traps according to *q* = 1, with pitfall traps being the least effective trap. Results from this study also support the use of multiple sampling techniques targeting different beetle taxa^42^, depending on the aims and objectives of the specific study.

Gunung Belumut is dominated by igneous rocks exposed to weathering and erosional processes^43^. Gunung Besar Hantu formed through volcaniclastic rocks in association with siliciclastic sediments of the Dohol Formation, according to Surjono et al.^44^. GBL is a pristine ecosystem with a good number of benthic macroinvertebrates^45^, whereas GBH possesses a high number of rove beetles that decompose animal and plant materials found in the forest reserve^46^. Endemic species contributed most to single mountain species richness in this study, likely due to a number of reasons including the biogeographical history of the mountain, climatic influences, and dispersal ability of species and their niche preferences. The climatic niche widths will restrict the species within a narrow range of elevations, leading to increased isolation, which promotes endemism and ultimately speciation^47^. We detected high biotic heterogeneity among all mountains, where comparison of Gunung Inas with Cameron Highland, Chamah Highland, Gunung Benom, Gunung Angsi, and Gunung Besar Hantu have high turnover values, which means that the identity of the species differs among Gunung Inas and the other named mountains. Some species were shared among the six abovementioned mountains, including *Orthogonius asiatictus, Nodostoma sp1, Lacon sp1, Melanothus sp1, Pyrocoelia sp2, Crossotarus saundersi, Anomala cupripes, Anomala grandis, Apogonia sp1, Luperodes bimaculatus, Hyphasis* sp1*, *etc*.*

Indicator species analyses showed that beetle altitudinal and habitat diversity patterns depended on the biogeography of the mountain, phytogeographical patterns, local habitat choice by species, and climatic factors associated with the mountains^48,49^. Highest indicator values were shown by *Coccotrypes variabilis* (65%), *Canthydrus haagi* (60%) and *Dischisus notulatus* (54%) but the biology of these species is very poorly known. Genus *Coccotrypes* is well adapted to warm and dry areas in the tropical forests of Asia and Africa where they feed and breed in small seeds, in particular palm seeds. Moreover, *Coccotrypes* is the only bark beetle genus known to breed in ferns^50^. The high indicator value here could be due to specific habitat affinities and specificity of the particular mountain and the plant community affiliated with these mountains. Responses of several beetle functional groups are tightly linked with their feeding guilds, and overall composite habitat complexity. Although habitat preferences by beetle species may regularly mirror their scavenging behaviours, elucidation of the causal mechanisms underpinning the relationships between habitat complexity and beetles are critical for the development of general principles linking habitat, functional roles and diversity^51^. Moreover, indicators are very useful in management of different ecosystems such as montane and forest ecosystems as discussed by Cosovic et al.^52^.

Montane ecosystems with such high beta diversity are common in the tropics^53^, although relatively understudied in Southeast Asia. Anthropogenic impacts, biogeographical factors including environmental filtering, and/or historical mechanisms are likely shaping the replacement patterns that we detected among the mountains included in this study. Moreover, locally co-existing species represent a large proportion of the regional species pool with the limited dispersal abilities of beetles^54^, which is also important in shaping the biodiversity of these mountains.

Nestedness may reflect the number of niches available or occupied at different sites, or result from extinctions in poor quality sites as suggested by Legendre^55^. Nestedness and turnover are the two components that imply distinct ecological processes determining biodiversity patterns^56–58^. Moreover, da Silva et al.^59^ suggests that partitioning beta diversity into contributions of individual species and contributions of single sites could be more useful in general ecological, bioassessment, and conservation decision making.

Beta diversity comparisons (NMDS for nestedness and turnover) using richness and species turnover showed significant differences in the following comparisons: CH-CHH, GI-CHH, CHH-GA, CHH-GBL, CHH-GBH, CHH-GBA, and CHH-GT. These mountains are going through an important ecological process of ‘lowland biotic attrition’, which means that species die or move and are not replaced, since there is no source of species adapted to warmer conditions^1^. Moreover, Feeley and Silman^60^ argue that biotic attrition is highly pronounced in the hot tropical lowlands, since raised temperatures may exceed the observed tolerance levels of most extant species, though responses of lowland tropical communities to climate change are poorly understood^61^. Thus, we strongly suggest investigating this phenomenon in these Malaysian mountains would help us to further understand lowland biotic attrition.

Since mountain regions/ranges harbor high levels of endemic habitat specialist species, unique environmental conditions, and limited options for range shifts, it has been perceived that the species extinction rate will be comparatively higher in these regions^3^. In this sense, our results show that mountain heterogeneity preserves high beetle diversity, as mountains maintain high replacement between sites and within elevations. This coincides with the hypothesis that climate niche conservatism plays a role in the elevational species distributions in tropical montane ecosystems. Apart from this ecological explanation, historical factors, such as past climate change and biogeographical history, immigration, priority effects, and evolutionary mechanisms should also be considered, since these factors are regularly interwoven^47^.

Across these studied mountains, a crucial aspect that remains to be evaluated is current levels of connectivity among them, and what levels of change in this variable are acceptable for the future conservation of biodiversity. A landscape point of view is essential to understand how anthropogenic variables and mosaic environmental attributes influence beetle diversity patterns. We need to explore not only elevations within and across mountains, but also microhabitat conditions in order to develop robust and effective conservation strategies. This study strongly advocates the necessity of managing land-use patterns in montane ecosystems, as well as preserving the remaining montane biodiversity, for future sustainable development. Fragmented habitats, further separated by human-transformed land cover, significantly reduce availability of habitats and alter behaviors of various species^62^—a primary causal factor of terrestrial biodiversity loss. To conserve insect diversity in landscapes altered for human use, it is essential to measure not only alpha diversity, but also beta diversity to understand species replacement among landscapes. This knowledge will guide decision makers in formulating effective strategies for the design of protected areas and for human land use.

Future studies should focus on separating the beetle communities into functional groups, which would be useful for understanding some of the factors likely driving the observed diversity patterns, and their potential conservation implications^63^. We need to analyse other dimensions of the beetle biodiversity as functional and phylogenetic metrics to unravel the factors and mechanisms driving membership of these communities across these mountains.

## Methods

### Study sites

**Figure 4:**
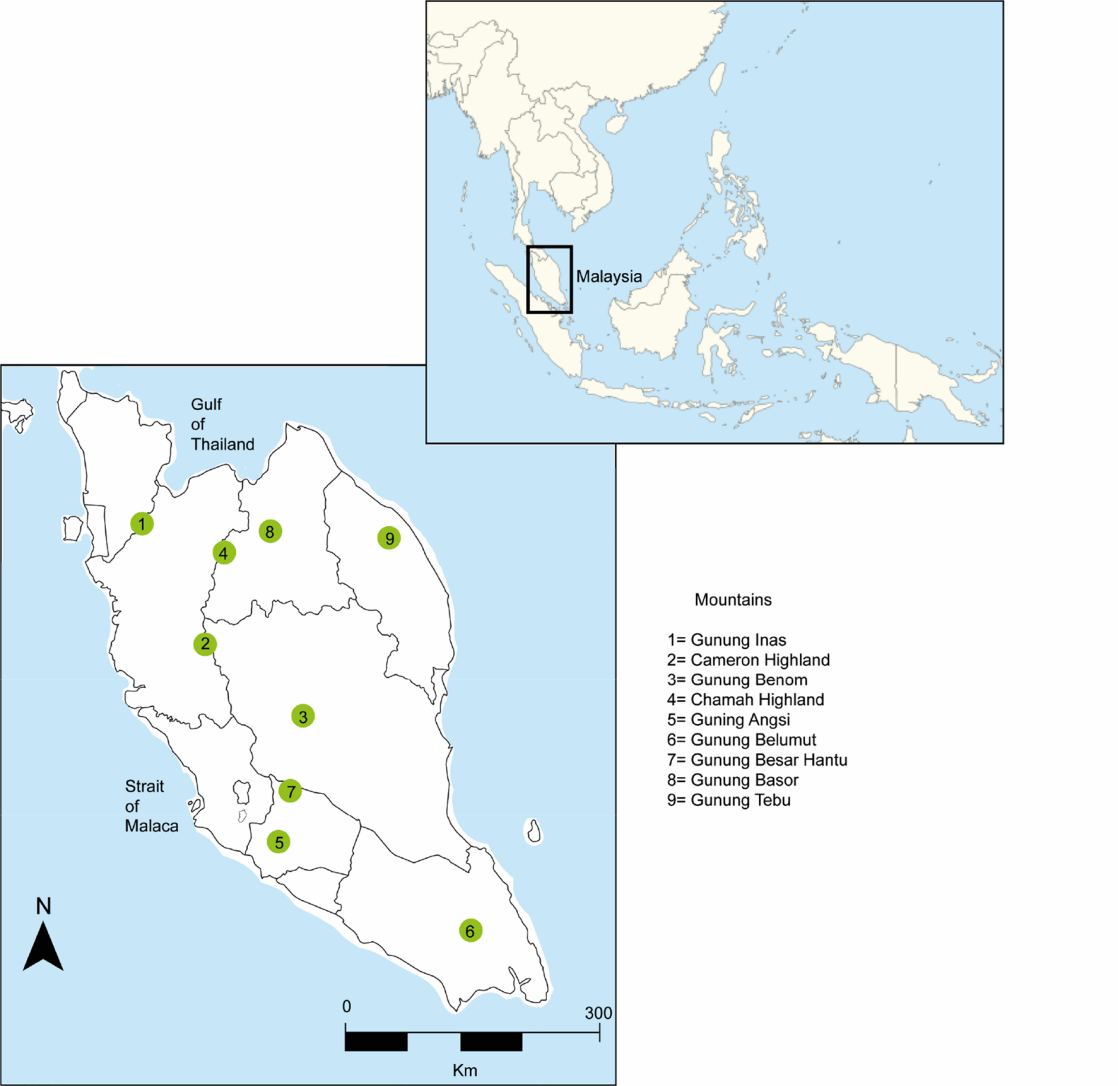
Mountains sampled for beetles in Peninsular Malaysia.

The sampled mountains were Gunung Inas (GI), Cameron Highlands (CH), Gunung Benom (GB), Chamah Highland (CHH), Gunung Angsi (GA), Gunung Belumut (GBL), Gunung Besar Hantu (GBH), Gunung Basor (GBA), and Gunung Tebu (GT) (Fig. 4). The coordinates of these mountains and their key features are listed on Table 3.

**Table 3:** Sampled Mountains and their special features relevant to this study.

Name of the mountain	GPS Coordinates	Special feature/s
Gunung Inas (GI)	5° 41′ N 100° 78′ E	Lowland dipterocarp, hill dipterocarp, lower montane and upper montane forests^64,65^
Cameron Highlands (CH)	4° 19′ N 101° 21′ E	Cameron Highlands is much cooler compared to lowlands in Malaysia, with a mean daily minimum of 14.8 °C and a mean daily maximum of 21.1 °C, which suits temperate crops^67^
Gunung Benom (GB)	3° 49′ N, 102° 5′ E	Gunung Benom has been considered a pristine zone with minimal anthropogenic interventions, with the major forest types found in this area being lowland, hill and montane forests with a unique assemblage of plant species^66^
Chamah Highland (CHH)	5° 13′ N 101° 34′ E	Remote and secluded mountain found in Kelantan state with dense forest and very limited human influences^31^
Gunung Angsi (GA)	2° 69′ N 102° 05′ E	Part of Ulu Bendol Recreational Forest in Negeri Sembilan State, which consists of 143 ha of virgin forest and is surrounded by approximately 360 ha of logged forests (logging was actively carried out from 1959 until 1977)^69^
Gunung Belumut (GBL)	2° 03′ N 103° 31′ E	Which is covered with highland dipterocarp forest type^71^
Gunung Besar Hantu (GBH)	3° 23′ N, 102° 012′ E	Which is covered by dipterocarp forest^70^
Gunung Basor (GBA)	5° 36′ N 101° 48′ E	Covered with lowland dipterocarp hill forest, upper dipterocarp forest and lower montane forest, where the dipterocarp forest has been selectively logged on several occasions since the 1970s^68^
Gunung Tebu (GT)	5° 34′ N 102° 33′ E	Extent of 25,529 ha in the state of Terengganu, which contains valuable timber species mainly from the families Dipterocarpaceae and Euphorbiaceae, with the former being the most dominant family^34^

### Sampling protocol

Beetle samples were collected at 500 m and 1000 masl elevations from Gunung Angsi, Gunung Belumut, Gunung Basor and Gunung Tebu only, due to the height of these mountains, whereas the rest of the mountains were sampled at 500 m, 1000 m and 1500 masl. Samples were collected from September 2012 to September 2016 in regular 6-month intervals, and the sampled months were September and March across study years. Three sampling methods were used to collect the beetles from these locations. The traps operated simultaneously at each elevation. At each elevation on each mountain, we fixed 20 pitfall traps, four Malaise traps and four light traps, where all the fixed traps were at least 200 m apart from each other and placed at least 200 m away from the nearest main road.

Malaise traps consisted of a nylon net connected to a collection jar, half filled with 70% ethanol and attached to a tree branch about one meter above the ground. Pitfall traps were plastic cups (diameter 65 mm, depth 95 mm) partly filled with 70% ethanol and dug into the ground with the rim flush with the soil surface. We placed large dry leaves above each trap to protect them from litter and rain. At each collecting date, Malaise and pitfall traps were set for 24 h, starting at 08:00 a.m. Light traps were made of mosquito netting with a 160-W mercury bulb connected to a portable Honda EU10i portable generator. At each collecting date, the light traps operated from 18:30 to 23:30, and beetles were collected manually from the traps using collection bottles and aspirators. We occasionally continued to use the light traps until the next morning until 06:00, but no beetles were captured after midnight.

### Identification of specimens

The collected beetles were sorted, summed and cross-checked using different keys^72–97^. Re-confirmation of the identified species was conducted at the collections of the Wildlife Department of Malaysia, University of Malaya, National University of Malaysia and Forestry Department of Malaysia.

### Data analysis

First, we checked the accuracy of the inventory by using the sample coverage estimator suggested by Chao and Jost^98^, which is a less biased estimator of sample completeness. Sample coverage has values from 0 (minimal completeness) to 100% (maximum completeness). We compared diversity between mountains, elevations and sampling method/trap type with Hill numbers ^*q*^*D*^99^ of order *q* = 0 and *q* = 1^100^. In this sense, *q* = 0 is a measure of the degree of difference in species richness value; that is, the relative difference in the number of species between communities and is not sensitive to abundances; *q* = 1 uses the exponential of Shannon’s entropy to estimate species diversity as effective diversity. Effective numbers of species are the numbers of species with the same abundance that theoretically can coexist in a community with the maximum evenness. The effective number of species can tell us the magnitude of difference when we compare two sites or landscapes. Thus, this measure has biological sense and their results are widely comparable among communities (Jost 2006, Moreno et al., 2017). For further information about equations, see Jost^99^. We compared ^*q*^*D* values by using confidence intervals at 95% for mountains, elevations and traps. The inventory completeness, and biodiversity estimations were calculated using R software 3.3.1^101^.

We calculated the indicator value IndVal^102^; using the indicspecies package^103^ in R software R 3.2.1^101^. The IndVal combines measurements of habitat fidelity (frequency within that habitat type) and specificity (uniqueness to a particular site) to identify the characteristic species of each category with a value from 0 to 100%. Finally, the statistical significance of association was analysed using a permutation test between pairs of species and for groups of mountains using the multipatt function^104,105^.

The species composition was compared using a presence-absence similarity index (Jaccard) and abundance based index (Bray–Curtis similarity index). Differences were analysed with a permutational multivariate analysis of variance (PERMANOVA) after 999 permutations of residuals under the reduced model, as a non-parametric alternative to the multivariate analysis of variance^106^ for Jaccard and Bray–Curtis values for mountains and elevations. After PERMANOVA tests, pairwise tests were applied to determine differences in habitat pairs. Significant differences were set at P ≤ 0.05. These differences among samples for each mountain and elevation were represented in a NMDS (non-metric multidimensional scaling) with Bootstrap using the PRIMER v7 program^107^.

We partitioned beta diversity using the Baselga^108^ approach; according to this method, total dissimilarity (βcc) is 1 minus the similarity coefficient of Jaccard. This total dissimilarity is divided into two components: the dissimilarity due to turnover (species replacement between communities) (β.3) and the dissimilarity due to richness differences or nestedness (species gain or loss between communities) (βrich). This partition was done for the dissimilarity in the composition of species between the mountains with the R program^101^, with the script of Carvalho et al.^109^. The turnover component may occur because of environmental filtering or spatial and historical constraints^110^, but it will be independent of the differences in the number of species per site. The nestedness component of β-diversity is due to the fact that one assemblage is a subset of another.
